# Characterization of Aroma, Sensory Properties, and Consumer Acceptability of Honey from *Capparis spinosa* L.

**DOI:** 10.3390/foods14111978

**Published:** 2025-06-03

**Authors:** Gianluca Tripodi, Maria Merlino, Marco Torre, Concetta Condurso, Antonella Verzera, Fabrizio Cincotta

**Affiliations:** 1 Department of Human Sciences and Promoting of the Quality of Life, San Raffaele Telematic University Rome, Via Val Cannuta 247, 00166 Rome, Italy; gianluca.tripodi@uniroma5.it; 2 Department of Veterinary Sciences, University of Messina, Viale G. Palatucci, 98168 Messina, Italy; maria.merlino@unime.it (M.M.); or marco.torre@unito.it (M.T.); concetta.condurso@unime.it (C.C.); antonella.verzera@unime.it (A.V.); 3 Department of Agricultural, Forestry and Food Sciences—DISAFA, University of Turin, Via Verdi 8, 10124 Torino, Italy

**Keywords:** caper honey characterization, aroma volatiles, sensory descriptors, consumer’s acceptability

## Abstract

The increasing scarcity of traditional nectar sources due to climate change has led beekeepers to explore alternative floral sources. This study investigates the volatile profile, sensory characteristics, and consumer acceptability of monofloral honey derived from *Capparis spinosa* L., a drought-resistant Mediterranean plant. Honey samples produced by *Apis mellifera* ssp. *sicula* on Aeolian Islands (Sicily, Italy) were analyzed. Volatile organic compounds (VOCs) were extracted using headspace solid–phase microextraction (HS-SPME) and identified by gas chromatography–mass spectrometry (GC–MS), revealing 59 compounds, with dimethyl sulfide being the predominant one. Sensory evaluation using quantitative descriptive analysis (QDA) and Time Intensity (TI) analysis identified distinctive descriptors such as sweet-caramel, cabbage/cauliflower, and pungent notes. Statistical analyses confirmed correlations between specific VOCs and sensory perceptions. A consumer acceptability test involving 80 participants showed lower preference scores for caper honey in terms of aroma and overall acceptability compared to commercial multifloral honey, with differences observed across age groups. The unique aromatic profile and consumer feedback suggest that caper honey has strong potential as a niche, high-quality product, particularly within the context of climate-resilient beekeeping, offering valuable opportunities for innovation and diversification in sustainable apiculture.

## 1. Introduction

In recent years, the beekeeping sector has been facing increasing challenges due to the adverse effects of climate change. The rising temperatures, prolonged droughts, and shifting blooming periods are significantly affecting the availability and productivity of many floral sources traditionally used in apiculture. As a result, there is a growing interest in the identification and valorization of alternative nectar sources, particularly those derived from plants that are resilient to climate change.

*Capparis spinosa* L., commonly known as the caper plant, is a perennial shrub belonging to the Capparaceae family. It is a typical species of the Mediterranean ecosystem, widely distributed across arid and semiarid regions of southern Europe, North Africa, and Central Asia [1,2]. The caper plant is characterized by its remarkable drought resistance and the ability to thrive under high temperatures, making it well adapted to increasingly arid environments and a valuable nectar source during the summer months, when other floral resources are scarce. Moreover, it has been traditionally used in herbal medicine for its high amount of bioactive compounds, such as polyphenols, alkaloids, glycosides, tannins, and flavonoids [3,4,5,6]. In this regard, several studies have demonstrated its antimicrobial, antidiabetic, anti-inflammatory, and antioxidant properties [7,8,9,10].

In Italy, the caper plant is mainly found in Sicily, particularly along coastal areas and nearby small islands, with scattered populations occurring inland. Traditionally, *C. spinosa* is cultivated for its flower buds (capers) and fruits (caper berries), both widely used as condiments in culinary preparations. However, the reduction in pollen availability from Mediterranean plants such as citrus, whose production has declined by approximately 18% [11], has led honeybees to shift toward more drought-tolerant native species. In this context, *Capparis spinosa* L. has attracted growing interest as a resilient nectar source, leading to growing attention on the production of caper monofloral honey.

However, caper honey has been the subject of a limited number of studies, primarily focused on its antioxidant and antibacterial activities, as well as its potential immunomodulatory effects, suggesting that it could represent a valuable functional food [12,13,14,15].

Honey is widely recognized for its health properties due to its natural antioxidant content and associated health benefits [16]. Its chemical composition is strongly influenced by the floral source, geographical origin, environmental conditions, and post-harvest handling [17,18,19]. Among its components, volatile organic compounds (VOCs) are of particular importance, as they play a key role in determining the aroma and taste of honey and are commonly used to authenticate the botanical origin [20].

However, despite these promising findings, no studies to date have explored the chemical and sensory properties of caper honey. In particular, no data is available on its volatile composition or consumer acceptability, both of which are essential for evaluating its potential as a high-quality niche product in the honey market.

Considering the above, the present study aims to investigate the volatile profile of caper honey. Furthermore, a sensory evaluation was carried out to assess its sensory properties. This dual approach is expected to provide valuable insights into the functional and commercial potential of caper honey in the context of climate-resilient beekeeping.

## 2. Materials and Methods

### 2.1. Sampling

Caper honey samples produced by the endemic Sicilian black honeybee (*Apis mellifera* ssp. *sicula*) were provided by a local beekeeper from Aeolian Islands (Aeolian Archipelago, Sicily, Italy), who guaranteed their authenticity and botanical origin. A total of eight samples, packaged in glass containers, were collected from the 2023 and 2024 production years. The samples were transported to the laboratory, where they were stored at room temperature and protected from light until analysis. A commercial, multifloral honey was also used as a reference in the consumer’s acceptability test.

### 2.2. HS-SPME Conditions

The extraction of volatile compounds from honey samples was carried out by headspace solid-phase microextraction (HS-SPME). A sample of 5 g of honey was placed into a 40 mL vial, with the addition of 15 mL of NaCl saturated aqueous solution. Extraction was performed in the headspace vial kept at 40 °C using a Divinylbenzene/Carboxen/Polydimethylsiloxane (DVB/CAR/PDMS) fiber of 50/30 µm film thickness (Supelco, Bellefonte, PA, USA) and housed in its manual holder (Supelco, Bellefonte, PA, USA). The extraction times consisted of an equilibration time of 30 min and an extraction time of 30 min, during which the sample was constantly stirred. Following sampling, the SPME fiber was placed into a GC injector operating in splitless mode at 260 °C and maintained for 3 min to allow thermal desorption of the analytes onto the GC capillary column.

### 2.3. GC-MS Analyses

The GC-MS analyses were carried out using a Shimadzu GC 2010 Plus gas chromatograph, which was interfaced with a TQMS 8040 triple quadrupole mass spectrometer (Shimadzu, Milan, Italy). For the separation of the volatile compounds, a VF-WAXms, 60 m, 0.25 mm i.d., 0.25 μm film thickness polar column (Agilent Technologies Italia S.p.A.; Milan, Italy) was used. The analytical parameters were set as follows: injector temperature, 260 °C; injection mode, splitless; oven temperature, 45 °C held for 5 min, then increased to 80 °C at a rate of 10 °C/min, and then to 240 °C at 2 °C/min; carrier gas, helium at a constant flow rate of 1.0 mL/min; transfer line temperature, 230 °C; ionization technique, electronic impact (EI) at 70 eV; acquisition range, 30–400 *m*/*z*; scan speed, 1428 (amu/s).

Each compound was identified by comparing mass spectral data with NIST’ 24 (NIST/EPA/NIH Mass Spectra Library, version 2.0, Gaithersburg, MD, USA) and FFNSC 3.0 database (Shimadzu, Kyoto, Japan), and linear retention indices (LRIs) calculated according to the Van den Dool and Kratz equation. Where available, compounds were further confirmed by the injection of analytical standards (Sigma Aldrich, Milan, Italy) [21]. Quantitative results were obtained from total ion current (TIC) peak areas (average of three replicates) and expressed as area percentage.

### 2.4. Sensory Analysis

Qualitative descriptive analysis (QDA) was performed on caper honey samples, following the ISO standard procedures for panel selection and judge training protocols [22]. A sensory panel consisting of 8 trained judges (4 males and 4 females) aged between 22 and 60 years, was recruited among the personnel of the University of Messina. The panelists were selected and trained in agreement with Ferreira et al. (2009) [23]. The participants have given their written consent according to the principles of the Declaration of Helsinki. The subjects did not experience any risk as a result of the sensory test. The judges signed a consent form to undergo the sensory analysis.

The panel was trained through targeted sessions using commercial honey reference samples and a list of 20 sensory attributes related to appearance, odor, taste, and texture was initially developed. Based on the frequency of citations, 16 descriptors including 2 visual descriptors (color intensity; crystallization), 7 odor descriptors (sweet-caramel; floral; fruity/citrus; woody; balsamic; pungent; cabbage/cauliflower), 6 taste descriptors (sweet; acid; bitter; salty; caper; astringent) and 1 for texture (viscosity) were selected. Subsequently, a common vocabulary was established to define and standardize the sensory descriptors, ensuring consistent interpretation. Each descriptor was thoroughly explained and discussed to eliminate ambiguity and to familiarize the panelists with the evaluation scales and procedures. During the three hours before the test sessions, participants refrained from drinking (except water), eating, and smoking.

The sensory analysis was conducted by evaluating the intensity of the descriptors on a scale from 1 to 9, where “1” indicated no perception and “9” a very intense perception. Two samples were analyzed during each test session. Between each sample, the palate was restored with water. Each sample was assessed in triplicate.

### 2.5. Time Intensity Analysis

Four odor descriptors (cabbage/cauliflower, pungent, floral, sweet-caramel) and one taste descriptor (sweet taste) used in QDA analysis were selected for the Time Intensity (TI) analysis. Training sessions and evaluation procedures were conducted according to the method proposed by Sokolowsky et al. (2015) [24].

The evaluation was stopped automatically after 180 s or individually by the judges when no more intensity was perceived. Data were collected at 0.5 s intervals. For each session, two honey samples were presented in a randomized order. Time Intensity was calculated according to Sokolowsky et al. (2015) [24], obtaining TI curves.

### 2.6. Consumer’s Acceptability Test

The consumer’s sensory acceptability of caper and multifloral honeys was evaluated by a panel of 80 untrained judges, 36 males and 44 females, ranging from 20 to 69 years old, and recruited from the Department of Veterinary Sciences (University of Messina, Messina, Italy) among habitual honey consumers. All participants involved in the study signed a written consent in alignment with the ethical standards established by the Declaration of Helsinki.

Panelists were asked to evaluate the appearance, aroma, taste, and texture of honeys.

Samples were identified with three-digit codes and served randomly on white teaspoons. After each sample was tasted, water was consumed to restore the palate. The participants expressed their judgements using a 9–point hedonic scale (1 = dislike extremely, 2 = dislike very much, 3 = dislike moderately, 4 = dislike slightly, 5 = neither like nor dislike, 6 = like slightly, 7 = like moderately, 8 = like very much, and 9 = like extremely) [21]. The overall acceptability was determined using the average value of the above parameters. In addition, participants were asked to answer the questions, “Would you consume this product?” and “Would you buy this product?” with “yes” or “no”.

### 2.7. Statistical Analysis

The XLStat software, version 2024.1 (Addinsoft, New York, NY, USA), was used to statistically analyze the GC-MS and sensory data. Pearson’s correlation analysis was conducted to correlate GC and sensory data. One-way Analysis of Variance (ANOVA) and Duncan’s multiple range test (significance defined as *p* < 0.05) were applied to sensory acceptability data to determine significant differences among caper and multifloral honey samples. Bonferroni correction has been applied for multiple comparisons.

## 3. Results and Discussions

### 3.1. Volatile Aroma Profile

The volatile aroma profile of caper honey showed a complex and unique chemical composition among unifloral honeys, constituted by 59 volatile compounds including acids, alcohols, aldehydes, ketones, aromatic, furanoic, sulfur compounds, and terpenes (Table 1).

Among the detected volatiles, dimethyl sulfide, a sulfur compound, was the most abundant, accounting for 59.34 ± 0.05% of the total volatile fraction. This compound, typically associated with boiled cabbage or vegetal odors, may derive from the enzymatic degradation of sulfur-containing amino acids present in caper nectar and suggests it plays a critical role in the honey aroma profile. The dominance of sulfur compounds, including dimethyl sulfide, is uncommon in most monofloral honeys, and such high abundance could be considered a chemical marker for caper honey. However, similar sulfur-containing volatiles have been identified in honeys from certain botanical sources. For instance, in a study analyzing monofloral honeys from the Brazilian semiarid region, sulfur compounds were among the characteristic volatiles, contributing to the unique aroma profiles of those honeys. This suggests that the presence of sulfur compounds can be a distinguishing feature in honeys derived from specific floral sources [31]. Thus, the predominance of this sulfur compound in caper honey may be linked to its specific botanical origin; in fact, capers (*Capparis spinosa* L.) are known for producing several sulfur metabolites that characterize the flower buds and fruits’ aroma profile [32,33,34]. However, despite the high concentration in the caper honey matrix and its odor threshold (2.6 µg/kg), the sensory impact of dimethyl sulfide is relative, likely influenced by its high volatility and interaction with other aroma compounds [35].

Acids accounted for 11.59 ± 0.15% of the total volatiles, with 2-propenoic acid (5.02 ± 0.03%) and nonanoic acid (2.02 ± 0.02%) being the most abundant. Their presence is consistent with previous studies on Mediterranean honeys, where medium-chain fatty acids contribute to the honey’s pungent and slightly rancid aroma notes [36]. In contrast to typical multifloral honeys, caper honey displays a higher concentration of longer-chain acids, such as dodecanoic acid (0.51 ± 0.02%) and decanoic acid (0.45 ± 0.01%) [37].

Aldehydes (13.06 ± 0.30% of the volatile profile) with nonanal (3.09 ± 0.04%), decanal (2.11 ± 0.03%), and hexanal (2.99 ± 0.03%) compounds are commonly associated with green, fatty, and citrus-like notes and are typically derived from lipid oxidation processes. The aldehyde profile of caper honey is particularly rich compared with other monofloral honeys, which tend to be dominated by monoterpenes or phenolic derivatives [38,39].

Among alcohols (4.78 ± 0.21%), 2-ethyl-hexanol (1.84 ± 0.03%), 1-octen-3-ol (0.67 ± 0.03%), and 1-dodecanol (0.63 ± 0.03%) were notable for their relatively high abundance. These long-chain alcohols are unusual in such quantities and may arise from wax-related degradation or enzymatic activity during honey ripening. Their contribution to aroma is generally mild but may influence mouthfeel and sweetness perception [40].

Aromatic compounds, especially benzaldehyde (4.37 ± 0.04%), were present in significant amounts. Benzaldehyde contributes to almond-like notes and has been reported as a prominent volatile in several monofloral honeys. Benzaldehyde is known as a floral marker in some honeys and is biosynthetically linked to phenylalanine metabolism. Moreover, the presence of furanoic derivatives such as furfural (2.41 ± 0.04%) supports the hypothesis of Maillard-type reactions occurring during honey processing or storage [41].

Terpenes, although present in lower concentrations (1.36 ± 0.12%), include compounds like linalool (0.30 ± 0.02%) and α-terpineol (0.10 ± 0.01%), which are valuable as floral markers. These compounds contribute to the floral aroma of honey and have been used in the classification of honey botanical origins [42].

Furanoic compounds were mainly represented by furfural (2.41 ± 0.04%). This class of compounds, which also includes 2-furanmethanol, 1-(2-furanyl)-ethanone, 2,5-dimethylfuran, 2-pentylfuran, 2-acetylfuranfurfural, furfuryl alcohol, and 5-methylfurfural, is usually found at trace levels in freshly harvested honey. Chemically, they are generated via the acid-catalyzed dehydration of pentoses, and their formation can be further enhanced by nonenzymatic browning reactions during thermal processing or prolonged storage [43]. Even at low concentrations, furfural is characterized by a sweet aroma that contributes to the overall flavor complexity of honey [44]. Its concentration increases during storage and thermal processing, with a higher concentration at higher temperatures. Consequently, these compounds are commonly used to assess the quality deterioration of honey. However, because their formation is primarily influenced by post-harvest processes rather than by the botanical origin of honey, they are not suitable as floral markers [45].

### 3.2. Qualitative Descriptive Analysis (QDA)

**Figure 1:**
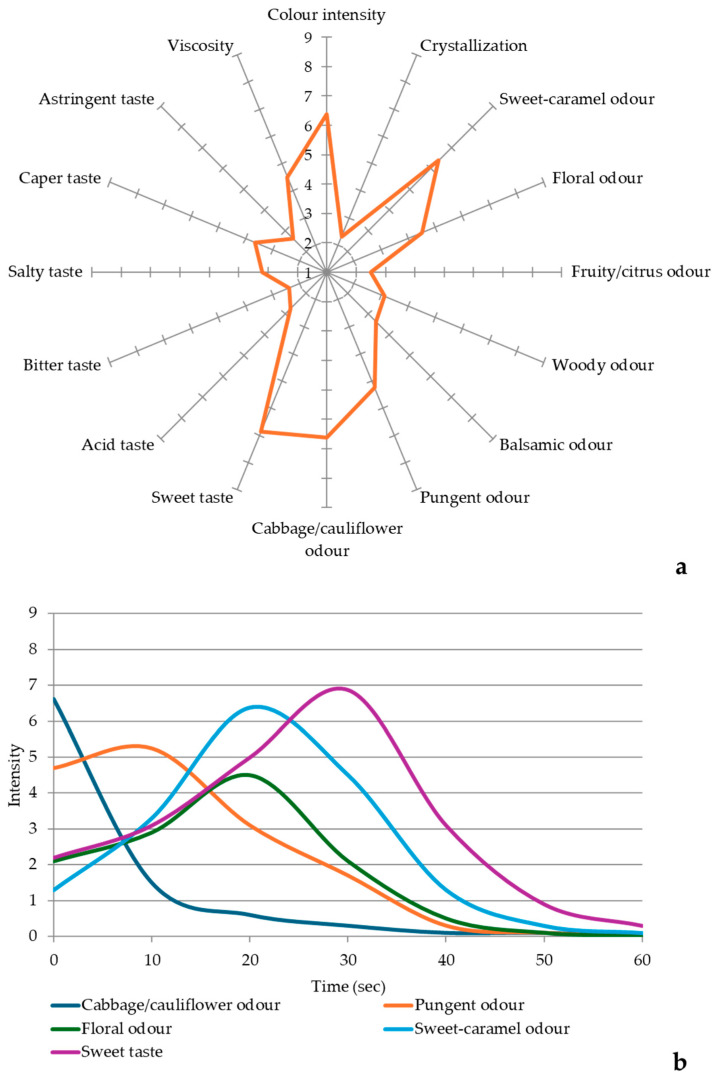
(**a**) QDA sensory plot of caper honey sample; (**b**) TI curves of the main sensory attributes of caper honey.

Figure 1a reports the graphical representation of the quantitative descriptive analysis (QDA) of caper honey evaluated by a trained panel.

Regarding its visual appearance, caper honey displayed a clear amber to light amber color with golden hues. It appeared clear and exhibited a low degree of crystallization. Its optical clarity indicates low levels of particulate matter and high-quality extraction practices. The low degree of crystallization is generally associated with the fructose/glucose ratio. Honey with a high fructose/glucose ratio (F/G > 1.3) typically resists crystallization, as glucose is less soluble in water and tends to crystallize easily [46].

The aroma of caper honey is distinctive and complex, dominated by moderately intense vegetal and floral notes characteristic of caper blossoms perceived as reminiscent of boiled cabbage and cooked onion. This is directly attributable to the high concentration of dimethyl sulfide (59.34 ± 0.05%), a compound known for its distinctive sulfurous aroma [33]. However, the perception of this note, although initially dominant, tends to fade over time, giving way to more pungent and sweet notes. This was assessed through the Time Intensity (TI) analysis, which allowed the identification of changes in the dominant sensory attributes over time during tasting. According to the TI curves (Figure 1b), the cabbage/cauliflower odor was the most intense during the initial 3 s, after which it rapidly declined and became almost undetectable. Its rapid decrease in perceived intensity is likely due to the high volatility and a low boiling point (37 °C) of dimethyl sulfide, which facilitates its quick dispersion at room temperature. Between approximately 3 and 14 s, the pungent odor became the dominant sensory note. This descriptor is primarily associated with the presence of organic acids, particularly 2-propenoic acid, identified in the caper honey samples.

The sweet-caramel notes identified by the panel were well supported by the presence of furfural (2.41 ± 0.04%) and other furanic compounds, such as furfuryl alcohol and 2-acetylfuran, commonly associated with Maillard reaction products [47]. Additionally, the woody and balsamic odor, as well as the citrus-like and sweet floral perceptions, were consistent with the presence of terpenes and aromatic alcohols such as linalool, *cis*-linalool oxide, and phenylethyl alcohol, albeit at lower concentrations (0.30 ± 0.02%, 0.50 ± 0.03%, and 0.11 ± 0.01%, respectively). These compounds have been reported to significantly contribute to the sensory complexity and floral character of monofloral honeys [48].

In TI analysis, the sweet-caramel and floral odor descriptors were initially perceived at low intensity but became predominant after approximately 14–16 s. The floral note, associated with the presence of 2-ethylhexanol, benzaldehyde, and terpenes, reached its peak intensity at around 20 s. The limited intensity of floral notes is likely due to two main factors: the high boiling points of 2-ethylhexanol and benzaldehyde, and the low concentration of terpenes in the sample. In contrast, the sweet-caramel odor became the most prominent after 14 s and persisted until the end of the analysis. This perception is primarily attributed to the aldehyde content, identified as the second-most abundant class of compound in the samples, and furfural.

When tasted by the panelists, the honey was found to be moderately sweet, with a mildly astringent, salty, and caper taste. These attributes distinguish it from more conventional monofloral honeys. A medium viscosity characterized the mouthfeel. Concerning the taste sensation of sweetness, the TI curve showed a high perceptual intensity at 30 s. This perception, which is certainly due to the sugar content of the caper honey, is also influenced by volatile compounds, especially by furan compounds [37] and alcohols [40], which can influence the perception of sweetness.

Overall, the sensory expression of caper honey reflected a distinctive aromatic identity, characterized by a sulfur-dominated base, caramel warmth, and delicate floral overlays, making it markedly different from other monofloral honeys such as citrus, which are typically dominated by monoterpenes [49,50].

**Figure 2:**
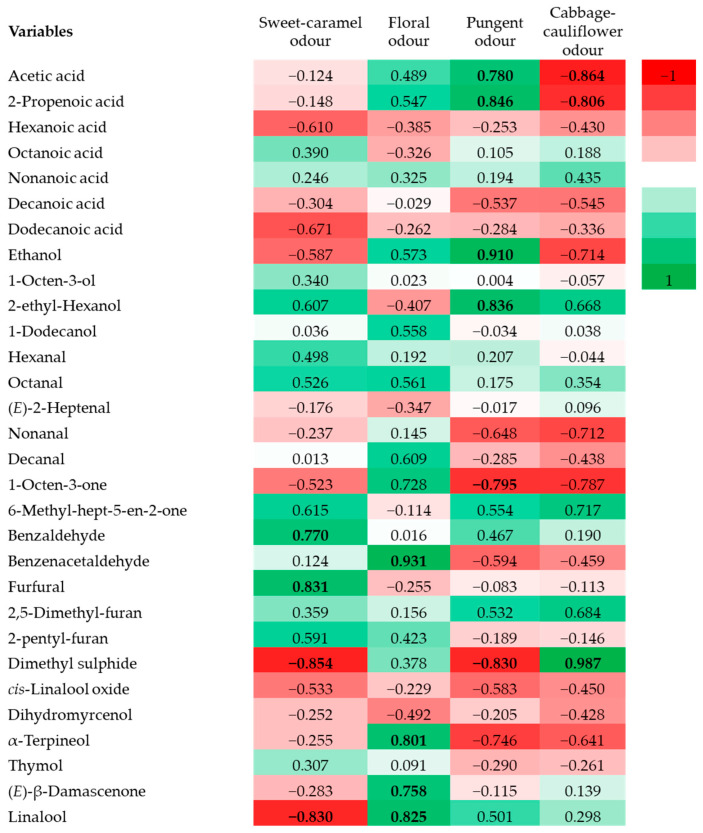
Pearson’s correlation heatmap of the main volatile aroma compounds and aroma sensory descriptors of caper honey. Correlations that have reached significance are indicated in bold.

The correlation heatmap between volatile compounds and sensory descriptors (Figure 2) highlighted that compounds such as benzaldehyde and furfural were strongly associated with the sweet-caramel odor of caper honey. In contrast, benzene acetaldehyde and terpenes such as α-terpineol, (*E*)-β-damascenone, and linalool were the major contributors to the floral aroma. The pungent odor appeared to be primarily linked to the presence of acetic acid, 2-propenoic acid, ethanol, and 2-ethyl-hexanol. Moreover, dimethyl sulfide showed a strong correlation with cabbage-like odor. These findings underscore the chemical complexity and aromatic distinctiveness of caper honey, reinforcing their potential as a valuable botanical and sensory marker for honey authentication assessment.

### 3.3. Consumer Acceptability

**Figure 3:**
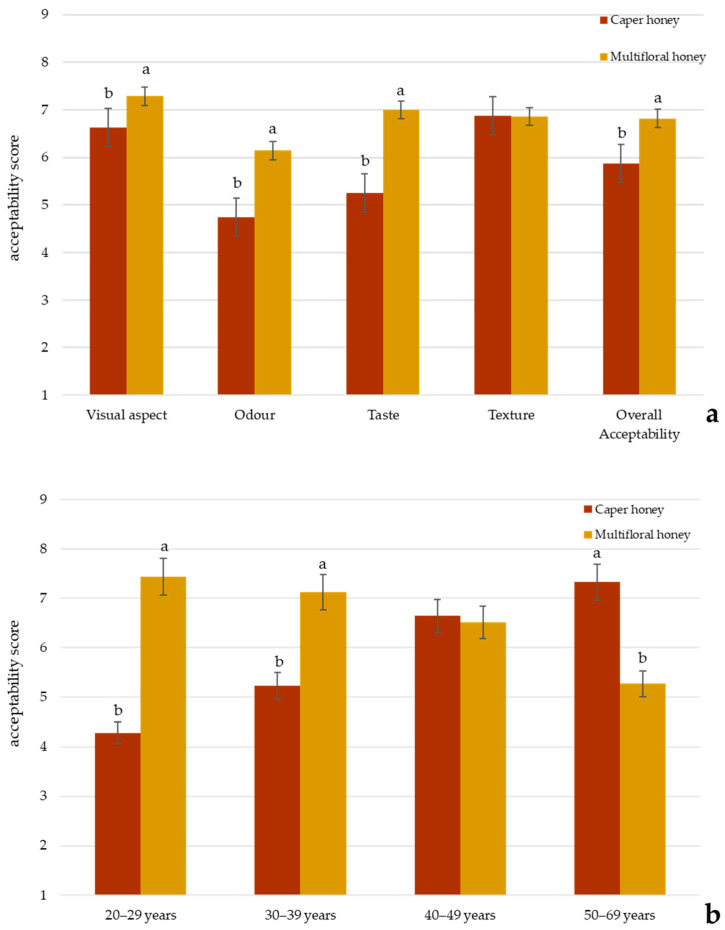
(**a**) Consumer acceptability of different attributes for caper and multifloral honey. Different letters for a specific sensory attribute indicate statistically significant differences at *p* < 0.05 by Duncan’s multiple range test; (**b**) comparisons of overall liking of caper and multifloral honeys as a function of age group. Different letters for a specific sensory attribute indicate statistically significant differences at *p* < 0.05 by Duncan’s multiple range test.

The consumer acceptability of caper honey was compared with a commercial multifloral honey by a panel of 80 untrained consumers. Sensory evaluation was conducted using a 9-point hedonic scale across five attributes: visual aspect, odor, taste, texture, and overall acceptability. Results (Figure 3a) indicated a statistically significant difference (*p* < 0.05) between the two samples for all the attributes except for texture, with multifloral honey receiving a higher mean score and an overall liking (6.72 ± 0.31) compared to caper honey (5.87 ± 0.43). The most pronounced difference was observed for taste, where multifloral honey scored significantly higher, indicating a greater consumer preference. Notably, caper honey had lower sweetness intensity, and the presence of salty and caper taste contributed to reduced acceptability among consumers preferring traditionally sweet honeys. Odor was the lowest-rated attribute for both honeys, but particularly for caper honey, suggesting a less favorable aromatic profile, probably due to its intense vegetable and pungent odor.

However, the demographic analysis (Figure 3b) revealed age-related differences in acceptability: younger participants (20–39 years) showed a significantly higher overall liking for multifloral honey, while older participants (40–69 years) demonstrated a greater appreciation for caper honey (*p* < 0.05). This could be attributed to the fact that older individuals may have greater culinary experience and greater familiarity with traditional or niche food products, leading to a more developed appreciation for distinctive odors and taste, such as that of caper honey. In contrast, younger consumers may be less familiar with such sensory attributes or may tend to prefer milder, sweeter odor and taste. Therefore, the observed age-related differences in acceptability likely reflect not only physiological factors but also sociocultural influences, including product knowledge, curiosity, and appreciation for atypical or complex sensory perceptions.

**Figure 4:**
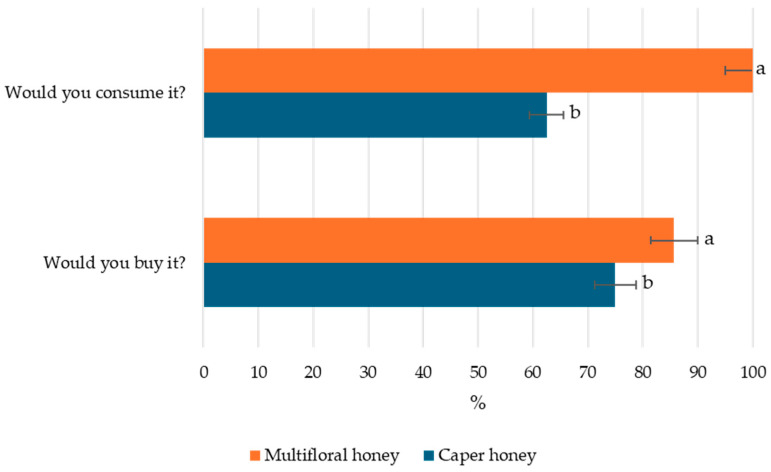
Consumption and purchase intent of caper honey compared with multifloral honey. Different letters for a specific sensory attribute indicate statistically significant differences at *p* < 0.05 by Duncan’s multiple range test.

Finally, Figure 4 reports the consumer responses regarding consumption and purchase intent for caper honey compared to multifloral honey. The results indicate a higher willingness to consume and purchase multifloral honey, with nearly 100% of participants expressing intent to consume it and over 90% indicating they would purchase it. In contrast, only approximately 65% of consumers reported a willingness to consume caper honey, and just under 75% would consider purchasing it.

These findings highlight the importance of consumer segmentation in product positioning, suggesting that the lower desirability of caper honey compared to multifloral honey, particularly regarding consumption intent, may be influenced by limited consumer awareness and its less familiar sensory profile, as evidenced by the sensory evaluation results. This further suggests that, while caper honey may have limited broad-market appeal due to its pungency and lower sweetness, its complex sensory profile and intense aromatic character make it well suited as a premium, niche product for gourmet markets and high-end culinary applications.

## 4. Conclusions

This study provides the first integrated characterization of caper (*Capparis spinosa* L.) honey, focusing on its volatile composition, sensory profile, and consumer acceptability. The honey demonstrated a highly distinctive volatile fingerprint, with an unusually high concentration of sulfur compounds (particularly dimethyl sulfide) alongside aldehydes, acids, alcohols, aromatic compounds, and a moderate presence of terpenes. This unique chemical composition is strongly influenced by the caper flower phytochemistry, suggesting its potential as a botanical authenticity marker.

The sensory evaluation revealed a peculiar sensory profile, combining vegetal odor of cabbage and cauliflower, floral, and sweet-caramel odors, as well as a moderate sweetness and astringent taste. Temporal Intensity analysis indicated a shift from pungent to floral and sweet sensations over time, contributing to its perceived complexity. Consumer’s acceptability test, while indicating lower overall acceptability compared to commercial multifloral honey, also highlighted significant appreciation among older consumers with higher inclination for artisanal and niche products. These findings underscore the potential for market segmentation and the positioning of caper honey as a niche gourmet product, with relevance in climate-resilient beekeeping and biodiversity preservation. Moreover, targeted marketing strategies emphasizing provenance, biodiversity, and sensory uniqueness could enhance consumer awareness and market value.

Future research should explore broader geographical sampling, long-term storage effects on volatile stability, and functional analyses to further validate the potential of caper honey as a high-value food product.

## Figures and Tables

**Table 1:** Volatile compounds identified in caper honey samples.

Compounds	LRI ^1^	Area %	Odor Descriptors ^2^
**Acids**			
Acetic acid	1446	0.62 ± 0.01	Vinegar-like
2-Propenoic acid	1627	5.02 ± 0.03	Acid, Tart
Hexanoic acid	1834	1.11 ± 0.02	Fatty, Green
2-Ethyl-hexanoic acid	1936	0.09 ± 0.01	Sweet herbal, Musty
Heptanoic acid	1943	0.35 ± 0.01	Plastic-like, Sweaty
Octanoic acid	2050	1.42 ± 0.02	Soapy, Fatty
Nonanoic acid	2160	2.02 ± 0.02	Soapy, Fatty
Decanoic acid	2272	0.45 ± 0.01	Rancid, Fatty
Dodecanoic acid	2486	0.51 ± 0.02	Rancid, Fatty
*Total*		*11.59 ± 0.15*	
**Alcohols**			
Ethanol	936	0.26 ± 0.01	Alcohol
1-Butanol	1141	0.06 ± 0.01	Medical, Phenolic
2-Methyl-1-butanol	1199	0.09 ± 0.01	Woody, Camphor-like
4-Methyl-1-pentanol	1292	0.01 ± 0.01	
2-Heptanol	1296	0.09 ± 0.01	Chemical
1-Hexanol	1330	0.13 ± 0.01	Balsamic, Aromatic herb
1-Octen-3-ol	1432	0.67 ± 0.03	Mushroom-like
1-Heptanol	1438	0.19 ± 0.01	Green
2-Ethyl-hexanol	1474	1.84 ± 0.03	Floral
1-Nonanol	1647	0.34 ± 0.02	Honey-like
Phenylethyl Alcohol	1900	0.11 ± 0.01	Rosy
1-Dodecanol	1957	0.63 ± 0.03	Fatty, Wax
1-Tetradecanol	2168	0.36 ± 0.02	Coconut
*Total*		*4.78 ± 0.21*	
**Aldehydes**			
2-Methyl-butanal	918	0.05 ± 0.01	Malty
3-Methyl-butanal	922	0.07 ± 0.02	Malty
Pentanal	984	0.27 ± 0.02	Almond, Malty, Pungent
Hexanal	1080	2.99 ± 0.03	Herb
(*E*)-2-Methyl-2-butenal	1098	0.33 ± 0.03	Green, Fruit
Heptanal	1177	0.28 ± 0.02	Green, Potato-like
2-Hexenal	1213	0.05 ± 0.01	Green, Leaf
Octanal	1272	1.43 ± 0.03	Soapy, Citrus-like
(*E*)-2-Heptenal	1309	1.64 ± 0.03	Soapy, Fatty, Almond -like
Nonanal	1379	3.09 ± 0.04	Fatty, Citrus-like
(*E*)-2-Octenal	1419	0.60 ± 0.02	Fatty, Soapy
Decanal	1488	2.11 ± 0.03	Lemon-like, Soapy
Dodecanal	1703	0.15 ± 0.01	Fruity
*Total*		*13.06 ± 0.30*	
**Ketones**			
4-Methyl-3-penten-2-one	1131	0.04 ± 0.01	Sweet, Chemical
2-Heptanone	1175	0.11 ± 0.02	Fruity
6-Methyl-2-heptanone	1225	0.02 ± 0.01	
2-Octanone	1267	0.02 ± 0.01	Soapy, Gasoline
1-Octen-3-one	1283	0.42 ± 0.03	Mushroom
2,5-Octanedione	1303	0.11 ± 0.01	
6-Methyl-5-hepten-2-one	1319	0.71 ± 0.03	Fruity
*Total*		*1.43 ± 0.12*	
**Aromatic compounds**			
Benzaldehyde	1517	4.37 ± 0.04	Almond-like
Benzene acetaldehyde	1638	0.95 ± 0.03	Honey-like
Toluene	1042	0.08 ± 0.01	Chemical
1-Methyl-3-(1-methylethenyl)-benzene	1426	0.07 ± 0.01	
*Total*		*5.47 ± 0.09*	
**Furanoic compounds**			
2-Furanmethanol	1651	0.07 ± 0.01	Sweet, Caramel, Burnt
Furfural	1456	2.41 ± 0.04	Sweet, Bread-like
1-(2-Furanyl)-ethanone	1501	0.03 ± 0.01	Candy, Caramel-like
2,5-Dimethyl-furan	956	0.06 ± 0.01	
2-Pentyl-furan	1216	0.26 ± 0.03	Green bean, Butter
2-Acetyl-furan	1499	0.08 ± 0.01	Candy, Caramel-like
*Total*		*2.91 ± 0.11*	
**Sulfur compounds**			
Dimethyl sulfide	754	59.34 ± 0.05	Sulfur, Vegetable, Boiled Cabbage
Dimethyl disulfide	1073	0.06 ± 0.01	Sulfur, Vegetable, Boiled Cabbage
*Total*		*59.40 ± 0.06*	
**Terpenes**			
*cis*-Linalool oxide	1427	0.50 ± 0.03	Fresh, Sweet, Floral
Dihydromyrcenol	1451	0.15 ± 0.01	Lime, Citrus, Cologne
α-Terpineol	1683	0.10 ± 0.01	Musty, Citrus
Thymol	2176	0.09 ± 0.01	Thyme
(*E*)-β-Damascenone	1805	0.10 ± 0.01	Honey-like
*trans*-Anhydrolinalool	1228	0.04 ± 0.01	Fresh, Sweet, Floral
*p*-Cymene	1253	0.04 ± 0.01	Solvent-like
Tetrahydro-Linalool	1413	0.04 ± 0.01	Wood, Citrus, Camphor
Linalool	1532	0.30 ± 0.02	Flowery, Sweet, Fruity
*Total*		*1.36 ± 0.12*	

^1^ Linear retention index. ^2^ Odor descriptors were identified by Flavornet Database https://www.flavornet.org/index.html (accessed on 5 May 2025) and references [25,26,27,28,29,30].

## Data Availability

The data are contained within the article.

## Abbreviations

The following abbreviations are used in this manuscript:

VOCs	Volatile organic compounds
HS-SPME	Headspace solid-phase microextraction
DVB/CAR/PDMS	Divinylbenzene/Carboxen/Polysimethylsiloxane
GC-MS	Gas chromatography–mass spectrometry
TIC	Total ion current
EI	Electronic impact
LRIs	Linear retention indices
QDA	Qualitative descriptive analysis
TI	Time Intensity Analysis
ANOVA	One-way Analysis of Variance
